# The role of adhesins of *Fusobacterium nucleatum* in colorectal cancer – a structural perspective

**DOI:** 10.1080/19490976.2026.2709265

**Published:** 2026-07-29

**Authors:** Felix Schöpf, Gian Luca Marongiu, Daniel Roderer

**Affiliations:** a Leibniz-Forschungsinstitut für Molekulare Pharmakologie (FMP), Berlin, Germany

**Keywords:** Colorectal cancer, bacterial adhesin, *Fusobacterium nucleatum*, structure-function relationship

## Abstract

*Fusobacterium nucleatum* is frequently found in the colon microbiome of colorectal cancer (CRC) patients. The bacterium is not only a passive bystander of CRC, but is actively involved in disease progression through mediating tumor growth stimulation, metastasis, and immune evasion. These outcomes are achieved through the action of several adhesins that are located on the outer membrane surface of *F. nucleatum*, which bind to different receptors on tumor or immune cells. Adhesin-receptor interaction as the initial step of host–pathogen interaction then triggers signal transduction pathways responsible for uncontrolled cell growth or downregulation of immune cells. For a number of CRC-relevant adhesins of *F. nucleatum* (FadA, Fap2, CbpF), the receptors have been known already for several years, but only recently mechanistic details have been elucidated through the determination of high-resolution structures of the complexes. In the case of other adhesins (RadD, Aim1), receptors have only recently been identified, or are as of yet unknown, and the mechanistic details underlying the interaction remain enigmatic. Here, we review the relevance of particular *F. nucleatum* adhesins in CRC progression, with a focus on recent mechanistic insights derived from structural biology.

## Introduction

In recent years, colorectal cancer (CRC) has ranked as the world's third most common cancer and the second deadliest, with over 1.9 million new cases and approximately 900,000 deaths, establishing it as an important global health problem. In particular for metastatic CRC, the prognosis is poor, with a 5-year survival rate of only 15%.^1^ ^,^ ^2^ The global incidence of early-onset colorectal cancer (EOCRC), defined as CRC in adults under 50, has been rising for decades and represents a concerning trend that has resulted in an increased mortality in this group.^3^ Overall, projections suggest a 72.5% worldwide increase in new cases by 2050.^4^ In light of these estimates, an understanding of factors contributing to the onset and progression of CRC is essential for the development of new strategies to mitigate the growing burden of this disease.

Dysbiosis, characterized by a disruption in the balance, diversity, and function of symbiotic microbial communities,^5^ is increasingly recognized as a contributing factor in CRC development.^6^ ^,^ ^7^ This disruption often manifests as shifts in the proportions of major bacterial phyla, the expansion of particular bacterial groups such as Proteobacteria, and an overall reduction in microbial diversity associated with an inflammatory environment.^8-10^ The increased risk of developing CRC in patients with inflammatory bowel disease (IBD), such as ulcerative colitis and Crohn's disease,^11^ ^,^ ^12^ is closely associated with gut dysbiosis and can typically be characterized by a reduction in microbial alpha diversity.^13-16^ For CRC, experimental data strongly suggest an important role of the microbiome, describing a specific community of microorganisms within a defined environment (microbiota) including their genomes and surrounding physicochemical conditions,^17^ in driving tumorigenesis: Transferring CRC-associated microbial communities from tumor-bearing mice into germ-free mice resulted in a rise in tumor burden, whereas the transfer from human donors to mice did not, which emphasized the importance of the initial microbial community composition on tumorigenesis.^18-20^ Meta-analyzes revealed global shifts in the composition of gut microbiota in CRC patients that are manifested by a decrease in butyrate-producing bacteria and an overabundance of bacterial species predominantly found in the oral cavity, such as members of the *Fusobacterium* genus.^21-23^

Fusobacteria are Gram-negative, anaerobic rod-shaped bacteria that are characterized by their strong capacity for adhesion and biofilm formation.^24^ ^,^ ^25^ The best described species of the genus, *Fusobacterium nucleatum*, is classified as an opportunistic pathogen that is associated with periodontitis and proliferates in dysbiotic oral environments. It potentiates the infectivity of other microbes and is therefore relevant to the disruption of host-microbial homeostasis.^26-28^ In addition to its role in the oral microbiome, *F. nucleatum* has also been found in intestinal environments, where it predominantly appears in conjunction with CRC.^29^ ^,^ ^30^ Moreover, *F. nucleatum* has been identified in esophageal or breast cancer,^30-32^ has been isolated from clinical specimens in cases of appendicitis, brain abscesses and pericarditis,^33-35^ and has been shown to be a causative agent in adverse pregnancy outcomes in mice.^36^ In the context of colorectal cancer, substantial evidence supports a driver role, where *F. nucleatum* actively participates in polymicrobial infections, promotes pro-inflammatory responses, and influences tumorigenesis through various mechanisms.^25^ ^,^ ^29^

The expression of multiple genes that encode proteins associated with adhesion and invasion enables *F. nucleatum* to bind and to invade diverse host cells, including colon epithelial and immune cells.^30^ ^,^ ^37^ ^,^ ^38^ It promotes biofilm formation by attaching to other microbes,^39^ ^,^ ^40^ stimulates the progression of CRC through various mechanisms mediated by its adhesins and lipopolysaccharides (LPS) present on the bacterial surface,^41-43^ and modulates the immune-response of the host, thereby facilitating immune evasion.^44^ ^,^ ^45^

*Fusobacterium nucleatum* is characterized by the absence of genes that encode for several protein secretion systems that are common in other Gram-negative bacteria, including the Tat system, the chaperone/usher pathway, and type I–IV systems, which typically facilitate environmental interactions.^46^ ^,^ ^47^ Instead, *F. nucleatum* utilizes the type V secretion system to transport a conserved family of large (200–400 kDa) autotransporter (AT) outer membrane proteins (OMPs) for adaptation to diverse host environments. The genome of *F. nucleatum* subspecies *nucleatum* (Fnn) ATCC 23726, for instance, encodes eight AT OMPs exceeding 200 kDa.^39^ ^,^ ^48^ The genomic abundance of AT adhesins is particularly pronounced in invasive *F. nucleatum* strains,^38^ ^,^ ^49^ which underlines their important role in pathogenesis.

**Figure 1.**
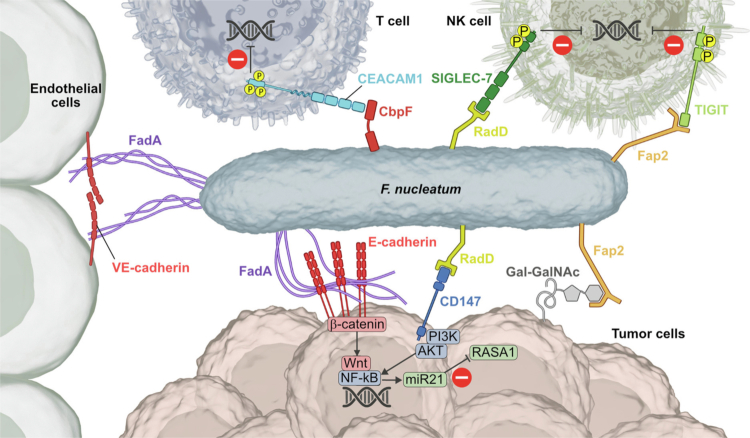
Overview of Fusobacterium adhesins relevant to CRC, their receptors on different cell types, and the subsequent intracellular regulatory pathways. FadA binds to VE-cadherin on endothelial cells and E-cadherin on tumor cells; the latter results in tumor cell growth stimulation through activation of Wnt/β-catenin signaling. Fap2 binds to the glycan Gal-GalNAc on tumor cells and to TIGIT on NK cells, the latter resulting in downregulation of their antitumor response. RadD binds to SIGLEC-7 on NK cells, resulting in their deactivation, and to CD147 on tumor cells, resulting in activation of PI3K–AKT–NF–κB–MMP9 signaling. CbpF binds to CEACAM1 on T cells, resulting in downregulation of their antitumor response.

Several adhesins mediate the bacterium's pro-tumorigenic effects. The most comprehensively described of these include the filament-forming Fusobacterium Adhesin A (FadA), and the AT proteins Fusobacterial Apoptosis Protein 2 (Fap2), Arginine-Inhibitable Adhesin D (RadD), and Carcinoembryonic Antigen Cell Adhesion Molecule (CEACAM) Binding Protein of Fusobacterium (CbpF)^41^ ^,^ ^43-45^ (Figure 1). FadA is the only adhesin described here that is not an AT protein. It binds to epithelial E-cadherin and vascular endothelial (VE)-cadherin, disrupting cell junctions and increasing vascular permeability, but also activating the *β*-catenin signaling pathway that results in the promotion of cell proliferation.^41^ ^,^ ^50^ The adhesin Fap2 binds to two different receptors on different cell types, promoting bacterial adhesion to tumor cells through interaction with D-galactose-β(1-3)-*N*-acetyl-D-galactosamine (Gal-GalNAc),^51^ and suppressing antitumor immunity through its interaction with the T-cell immunoreceptor with immunoglobulin and ITIM domains (TIGIT) on T and natural killer (NK) cells.^45^ ^,^ ^52^ RadD likewise exerts several functions, as it mediates interspecies interactions in biofilms,^39^ binds to the CD147 receptor on CRC cells, thereby resulting in the release of matrix metalloproteases that facilitate CRC progression,^43^ and inhibits NK cell mediated cancer cell killing through interaction with Siglec-7.^53^ The trimeric autotransporter adhesin CbpF engages the inhibitory receptors CEACAM1 and CEACAM5, which are expressed on various cells, including malignant cells and immune cells such as NK and T cells, resulting in the suppression of T cell effector functions.^44^ ^,^ ^54-56^

This review highlights how recent structural work has elucidated the molecular basis for the function of some of the fusobacterial adhesins, offering the first insights into their binding mechanisms at molecular detail. We also discuss limitations of the current structural data and the derived models, in particular those arising from the lack of detection of transient interactions. Moreover, it needs to be noted that structural biology delivers high-resolution snapshots of individual macromolecular complexes, typically outside their natural environment or in a highly simplified setting. These molecular snapshots require careful integration in the in vivo context to aid in interpreting complex functional data, in the described cases here for host–pathogen interplay that include, amongst others, bacterial attachment to cells, active invasion, secretion of metabolites, and activation of signal transduction cascades in the host.

### FadA

FadA was the first adhesin of *F. nucleatum* for which experimental high-resolution structural information was available.^57-59^ FadA is considered one of the main mediators of its adhesive properties, as FadA-deficient mutants exhibit drastically reduced binding to host cells.^57^ Beyond mediating cell adhesion, FadA plays a pivotal role in CRC progression by modulating host cell signaling. FadA binds specifically to E-cadherin on epithelial cells, disrupting cell–cell junctions, potentially facilitating tumor cell dissemination and metastasis, and activating Wnt/β-catenin signaling, which promotes oncogene expression and proliferation.^41^ Specifically in CRC cells, FadA also induces the expression of Annexin A1 (ANXA1), forming a positive feedback loop that further amplifies proliferation.^60^ In parallel, FadA-mediated activation of the E-cadherin/β-catenin pathway induces Chk2 expression and DNA damage, and promotes NF-κB–dependent inflammation and recruitment of tumor-supporting myeloid cells.^61^ ^,^ ^62^

FadA forms filaments that comprise two isoforms: the 129–amino-acid (aa) precursor preFadA, which contains an *N*-terminal 18-aa hydrophobic signal peptide, and the mature truncated 111-aa mFadA. Together, these two proteins assemble into the active complex FadAc,^63^ which is exposed on the surface of *F. nucleatum.* ^58^ ^,^ ^64^ To our knowledge, the formation of a functional protein complex by a non-cleaved precursor and the respective mature protein is unique to FadAc. While there are proteins whose precursor and mature forms have distinct biological functions, like interleukin-1a^65^ and Brain-derived neurotrophic factor,^66^ and proteins whose precursor and major isoforms form intermediate inactive complexes, like 13-16S proteasome precursor complexes,^67^ no complex represents the active, secreted form.

**Figure 2.**
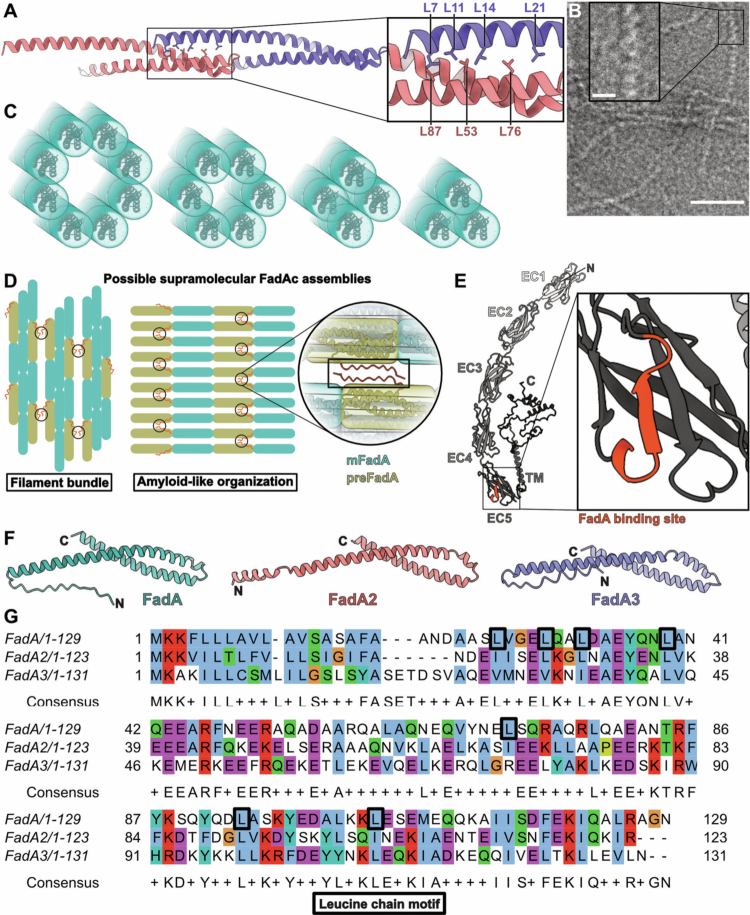
The adhesin FadA. A: Two mFadA subunits (PDB 3ETW) as organized in the filament. The inset shows the leucine chain motif. B: Negative stain EM micrograph that shows mFadA filaments. Scale bars: 50 nm, inset: 10 nm. Note that the filament diameter (ca. 9 nm) is considerably larger than linear individual FadA filaments as shown in A (ca. 3 nm), suggesting a supramolecular assembly. C: Possible FadA filament supramolecular assemblies of 8, 6, 4, and 3 individual filaments that would be compatible with the observed diameter of ca. 9 nm. D: The role of the signal peptide (dark red) in preFadA (yellow–green) in the assembly of FadAc. Lateral cross-linking of filament bundles (left) or an amyloid-like assembly (right) are conceivable, with the latter being incompatible with the supramolecular assemblies as shown in C. E: Predicted model of E-cadherin with five extracellular domains (EC1-5), the transmembrane region (TM), and the intracellular region. The FadA binding site as proposed in ref.^41^ is colored red. F: Models of FadA (PDB 3ETW, with predicted signal peptide) and its two homologs FadA2 and FadA3 (AlphaFold2 predictions). G: Structure-based sequence alignment of FadA, FadA2 and FadA3 from *F. nucleatum* subsp. polymorphum. Residues forming the Leu zipper in FadA are indicated. Note that not all of these leucines are conserved in FadA2 and FadA3.

X-ray crystallography has revealed that the mFadA monomer adopts a hairpin-like structure composed of two antiparallel *α*-helices connected by an eight-residue disordered loop.^58^ Along the Z-axis, mFadA monomers oligomerize in a head-to-tail fashion via hydrophobic leucine-leucine interactions. This interaction motif, termed the leucine chain, involves L7, L11, L14, and L21 of one monomer interacting with L53, L76, and L84 of an adjacent monomer (Figure 2A). Leucine residues also play an important role in the intramolecular stability of a hairpin monomer: L53 and L60 interact with L84 and L76, respectively.^58^

Intriguingly, electron microscopy analysis suggests significantly wider filaments than they would be expected from linear FadA assemblies (~9 nm vs. 3 nm, Figure 2B; own, unpublished observations), raising the question of how filaments organize at a higher level to form supramolecular assemblies (Figure 2C). Additionally, it has been shown that only FadAc, not mFadA, binds to E-cadherin on CRC cells and exerts pro-tumorigenic functions.^41^ ^,^ ^60^ This raises questions about the role of preFadA in determining the structure and function of FadAc. Qualitative evidence suggests that increasing proportions of preFadA promote the formation of shorter and wider filaments, as well as more knot-like structures, which in turn increased binding of FadA to CHO and HUVEC cells in culture.^59^ More recently, it has been reported that preFadA alters the FadA filament structure towards thicker, more bundled filaments, suggesting amyloid-like supramolecular organization.^64^ This shows that preFadA is not only a precursor to mFadA, but more importantly also acts as a structural and consequently functional modulator.

Since the only structural difference between preFadA and mFadA is the presence of the hydrophobic signal peptide, it has been hypothesized that interspersed preFadA monomers crosslink filaments into larger assemblies through hydrophobic interactions mediated by the *N*-terminus^64^ (Figure 2D). High-resolution structural data, e.g., cryo-electron tomography (cryo-ET), of connected FadAc filament bundles are required to illustrate the exact nature of these inter-filament crosslinks and the supramolecular assembly of FadAc.

In contradiction to the initial view that preFadA anchors FadAc to the inner membrane,^63^ FadAc assemblies were observed on the bacterial surface, indicating that preFadA must reach the extracellular space. Moreover, FadAc is expressed in the stationary phase, but not in the log phase, and has been found in periodontitis and CRC but not in healthy tissues,^64^ suggesting that FadA architecture and pathogenic potential may be regulated in response to environmental cues. Whether this regulation reflects controlled secretion of preFadA or results from spontaneous bacterial lysis, analogous to what has been described for bacterial toxins,^68^ remains unresolved.

The FadA host cell receptors have been identified as VE-cadherin and E-cadherin on endothelial and CRC cells, respectively.^41^ ^,^ ^50^ The dissociation constant (K_D_) to VE-cadherin was determined to 15.6 µM,^50^ a low-affinity interaction that is in the same range as bacterial adhesins targeting sugar moieties.^69^ In physiological contexts, however, multiple low-affinity interactions at numerous binding sites along a FadA filament could collectively generate high-avidity binding. This concept underlines the importance of FadA in host cell binding despite its low measured affinity, as evident from studies with FadA-deficient *F. nucleatum* mutants.^63^

Deletion studies mapped the FadA binding interface on VE-cadherin to the second half of extracellular (EC) domain 4 and first half of EC5.^50^ Later studies on the E-cadherin binding capacity of FadA narrowed down the binding site to an 11 aa stretch on the E-cadherin EC5^41^ (Figure 2E). Since E-cadherin forms *trans*-homodimers at cell–cell contacts between EC1 and EC2,^70^ FadA targeting EC5 enables *F. nucleatum* to bind E-cadherin molecules that are already engaged in cell‒cell contacts. This provides a structural explanation for the observed loosening of cell‒cell contacts, increased tissue permeability,^50^ and enhanced metastatic potential in the presence of *F. nucleatum* and/or FadA.^41^

So far, there is no solid structural evidence for the FadA side of the binding interface. It also remains elusive how the incorporation of preFadA into the supramolecular organization alters the unknown binding interface in a way that enables E-cadherin binding. It is conceivable that the FadAc supramolecular organization confers a level of molecular stability needed for effective binding to host cells without preFadA actually being part of the binding site, or that the interface is formed by several FadA subunits together, analogous to the actin‒myosin complex.^71^

Insights from whole-genome analyzes revealed that invasive *F. nucleatum* strains encode an expanded repertoire of FadA family homologs, including FadA2 and three copies of FadA3 (*fadA3a-c)* ^72^ (Figure 2F and G). Both Fnn strains ATCC 25586 and ATCC 23726 contain the genes for *fadA2 and fadA3a-c.* In contrast, *F. nucleatum subsp. polymorphum* ATCC12230 has a single copy of *fadA* and *fadA3.* ^72^ These homologs have been hypothesized to play a redundant role to canonical FadA or to expand the diversity of possible host receptors, but until now no mechanistic or structural evidence is available.

In summary, despite the existence of a structure of FadA and detailed functional analysis of the role of the preFadA precursor, critical gaps persist in understanding its supramolecular organization and implications on the interaction mechanism with the host. The assembly of FadA filaments into supramolecular bundles is unclear, as is the precise contribution of preFadA to this organization. Moreover, high-resolution structural information on the FadA/E-cadherin binding interface is required to elucidate the receptor binding mechanism. Cryo-EM can resolve filamentous and higher-order assemblies with near-atomic resolution,^73^ ^,^ ^74^ and thus is well-suited to dissect the architecture of FadA and its complexes with receptors. The weak affinity between FadA and cadherins, as determined for VE-cadherin,^50^ might, however, present an obstacle for an experimental co-structure at near-atomic resolution. Beyond isolated filaments, resolving the membrane-bound FadAc complex on intact *F. nucleatum using* cryo-ET will be essential to understand how membrane anchoring of the filaments is achieved. Moreover, Cryo-ET could be useful to overcome the aforementioned limitations arising from low-affinity FadA-E-cadherin interaction through examining FadAc filaments attached to target cell membranes. These structural insights would clarify how the regulation of the FadA supramolecular assembly affects the pathogenicity of *F. nucleatum* in CRC, and could potentially enable targeted therapeutic intervention against FadA-mediated tumor progression. Inhibition of supramolecular assembly has been carried out successfully for adhesive pili of uropathogenic *Escherichia coli,* ^75^ ^,^ ^76^ and should also be considered as a viable strategy to prevent fusobacterial adhesion to CRC cells via FadA.

### Autotransporter adhesins

Further CRC-relevant fusobacterial adhesins other than FadA are all secreted via the type V secretion system and are thus autotransporter proteins. Their extracellular domain is typically formed by the *N*-terminal part, whereas the C-terminus forms a *β*-barrel that anchors the protein in the OM. Different types of type V autotransporter proteins exist (subtypes a-f) and have been reviewed extensively elsewhere.^77^ CRC-relevant fusobacterial AT adhesins belong to the monomeric type Va subtype or the homotrimeric type Vc subtype, both of them remaining attached to the membrane. In the following, AT adhesins with known significance in CRC are outlined in detail, including known *F. nucleatum* strain-specific differences in receptor binding.

### Fap2

The adhesin Fap2 mediates fusobacterial adhesion to tumor epithelial cells by acting as a galactose-inhibitable lectin that binds to the Gal-GalNAc moiety, which is an overrepresented protein O-glycosylation on CRC cells.^51^ Fap2 also facilitates immune evasion of *F. nucleatum* by engaging TIGIT, which suppresses lymphocyte cytotoxic activity, induces apoptosis, and dampens antitumor immunity.^45^ ^,^ ^52^ The direct correlation between hemagglutination potency and TIGIT inhibition establishes a functional link between its lectin activity and immune-evasive properties, highlighting the bifunctional adhesin function of Fap2.^45^ In addition, Fap2 facilitates adhesion to *P. gingivalis* within the oral cavity, resulting in their coaggregation.^78^ Fap2 is the largest of approximately 2100 proteins encoded by Fnn ATCC 23726, and due to its unique sequence, it is identified as an outlier within the AT passenger family by sequence alignment analysis.^77^ ^,^ ^78^ The *fap2* gene (Fn1449) from Fnn ATCC 23726 (~11.4 kb) encodes the 3786-residue Fap2 (~400 kDa), which exhibits a canonical type Va AT architecture, comprising an *N*-terminal signal peptide (41 residues), a large extracellular passenger domain (3432 residues), and a C-terminal *β*-barrel domain (313 residues) that facilitates outer membrane anchoring.^48^ ^,^ ^79^ In Fnn ATCC 25586, the *fap2* gene was originally misannotated as a too short open reading frame coding for 3165 residues before it was annotated correctly to a 3738-residue ORF.^46^ ^,^ ^72^ In *F. necrophorum*, the invasive strain ATCC 25286 contains two Fap2 homologs, whereas *F. necrophorum* strain 1_1_36S does not have *fap2.* ^72^ In the CRC-associated *F. nucleatum subsp. animalis*, clade C1 does not contain *fap2* genes, whereas they are present in clade C2.^80^ Like many other AT proteins, Fap2 from Fnn ATCC 23726 and others lack cysteines, which is thought to prevent disulfide bond formation during periplasmic translocation.^49^

**Figure 3.**
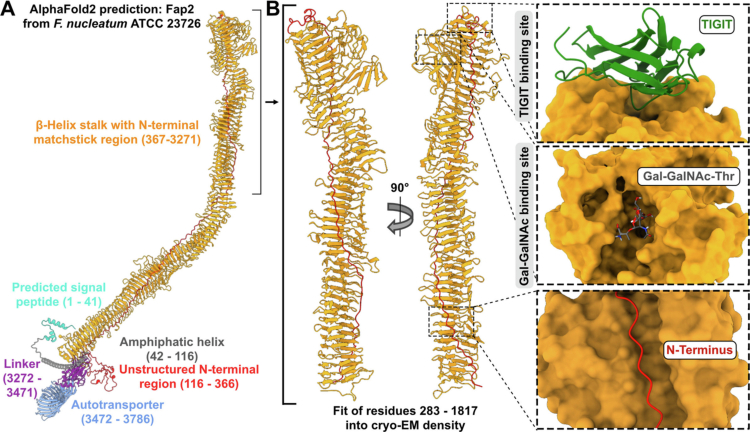
The adhesin Fap2. A: Full model of Fap2 of Fnn ATCC 23726, as shown in ref. ^79^ B: Model of the membrane-distal region (PDB 9QE7). The highlighted regions in the right panels show the *β*-helix in surface representation, while showing the models of docked TIGIT (top), docked Gal-GalNAc (center), and the *N*-terminus in the V-shaped cleft (lower) of Fap2 as ribbon or stick representations.

Recently, the structure of the Fap2 extracellular domain (Fap2-ECD) from Fnn ATCC 23726 has been solved with cryo-EM, which revealed an elongated, kinked, rod-like architecture of ~45 nm length.^79^ Its core is formed by a right-handed, three-stranded *β*-helix with a V-shaped cross-section. The *β*-helix is divided into two segments of 26 and 19 nm by a slightly variable kink (Figure 3A). The length and residue number of the Fap2-ECD place it among the largest known AT adhesins, far exceeding the 945 amino acid average for well-characterized AT passenger domains.^77^ ^,^ ^79^ ^,^ ^81^ The length of Fap2 demonstrates how pathogenic bacteria use extended polymeric structures to facilitate contact with host cells from a distance, enabling interactions beyond the bacterial capsule while avoiding immune responses that might be triggered by a short-distance adhesion.^82^

The Fap2 signal sequence comprises 41 amino acids, classifying it as a long signal peptide, as most ATs feature signal peptides of ~20 to 30 amino acids.^83^ This peptide exhibits the canonical two-domain organization of long signal peptides, featuring a 23-amino-acid, predominantly charged *N*-terminal extension followed by a hydrophobic C-terminal region. The presence of charged residues in the *N*-terminal region represents a conserved characteristic among these extended signal sequences.^84^ ^,^ ^85^

The V-shaped *β*-helix of Fap2 possesses a unique longitudinal hydrophobic groove, which is shielded by an unstructured, proline-rich amphiphilic *N*-terminal region that creates a hydrophilic surface (Figure 3A and B). This sequence motif is specific to AT proteins from the order Fusobacteriales, indicating its conservation among closely related species while being absent in more distant bacteria.^79^

Molecular docking suggested a distinct pit on the backside of the membrane-distal tip of Fap2 as the binding site for Gal-GalNAc (Figure 3B). Protrusions from the *β*-helix form the binding site, likely reducing solvent exposure and increasing the binding area to allow for more hydrogen bonds, analogous to the mechanism of buried mannose recognition sites that are employed by the *E. coli* lectin FimH.^86^ Molecular dynamics (MD) simulations showed a stable association of the ligand within this pocket, with hydrogen bond analysis implicating residues E619, R613, and F818 as primary contributors to the interaction. In cell-based assays, a Fap2 triple mutant (E619A/R613P/F818P) exhibited a reduction in its ability to adhere to cancer cells.^79^

Cryo-EM revealed that TIGIT, the immune cell interaction partner of Fap2, binds to its membrane-distal tip, supporting a model in which Fap2 neutralizes approaching immune cells from the greatest possible distance (Figure 3B). Integrative modeling suggested that Fap2 binds TIGIT at a different site than the poliovirus receptor (PVR), which is also supported by the observation that an antibody blocking the TIGIT-PVR interaction did not inhibit Fap2 binding to TIGIT.^45^ The interaction between Fap2 and TIGIT depends on TIGIT glycosylation. However, in the integrative model, both of the glycosylation sites of TIGIT (N32 and N101) faced away from the interaction site, suggesting an indirect involvement of the glycans.^79^ The ability of Fnn ATCC 23726 Fap2 to bind human but not mouse TIGIT,^45^ despite conserved glycosylation sites,^87^ further supports the hypothesis that the two glycans are not directly involved in the interaction interface. More high-resolution structural data are required to clarify the exact binding mechanism. Quantify analysis of the interaction of Fap2 with human TIGIT by surface plasmon resonance (SPR) analysis revealed a dissociation constant (*K*_D_) of ~0.6 µM,^79^ which lies between the interaction of TIGIT with its high-affinity ligand PVR (*K*_D_ ~1–3 nM)^88^ ^,^ ^89^ and its interaction with its lower-affinity ligand Nectin-2 (*K*_D_ ~6 µM).^90^

The species specificity of the Fap2/TIGIT interaction limits the relevance of mouse models to study the Fap2-mediated immunomodulation in vivo. As mouse and rat TIGIT share similar sequence identities to human TIGIT (59% and 58%, respectively, with ~85% identity between mouse and rat TIGIT), we expect that rat models are also not suitable, which eliminates the two most widely applied animal models for studying immunomodulation. Therefore, preclinical in vivo analysis for future pharmacological studies—for example, the analysis of Fap2 binding inhibitor candidates—must first identify suitable animal models.

The dual-receptor engagement by Fap2 is a pathogenic strategy used by various bacteria in order to facilitate host colonization and immune manipulation. For instance, a similar dual-receptor specificity is observed in the autotransporter UpaB from uropathogenic *E. coli*. Its structure also reveals two distinct binding sites on opposite faces of the protein: a groove that interacts with glycosaminoglycans and a polar region on the *β*-helix that binds host fibronectin.^91^ Another example is the Photorhabdus toxin complex (Tc), which binds to both glycosaminoglycans and Lewis antigens on target cells, proposedly in a two-step manner.^92^

In Fap2, the physical separation of approximately 3 nm between the unique and independent binding sites of TIGIT and Gal-GalNAc allows for the possibility to bind both receptors simultaneously, a capability that was shown for the pH 6 antigen (Psa) of *Yersinia pestis* and proposed for other bacterial adhesins such as the Dr adhesins from *E. coli* or the Multivalent Adhesion Molecule 7 (MAM7) widely expressed in Gram-negative bacteria.^93-95^ For Fap2, this is experimentally supported by a pull-down assay which demonstrated that Fap2-ECD retains its ability to bind TIGIT-ECD even in the presence of a large molar excess of GalNAc.^79^ This raises further questions about the interplay of both interactions *in vivo* and the physiological relevance of combining both functions on a single adhesin.

The existing Fap2 cryo-EM structure represents the first structural assessment of a large type Va autotransporter adhesin of *F. nucleatum*, but still numerous limitations remain for interpreting the molecular mechanisms of receptor binding and also for the potential design of an adhesion inhibitor. With ~4.5 Å resolution, the cryo-EM data of Fap2^79^ allowed fitting of an AlphaFold2 model, but no modeling of amino acid side chains. This will limit the applicability of the current model for *in silico* binding analysis. The proposed Gal-GalNAc binding mechanism is solely based on docking and MD simulation so far and still requires highly resolved experimental structural data for utilization in inhibitor design studies. The same is true for the Fap2/TIGIT interaction site, where the existing low-resolution structure only revealed the position of the binding site but no atomistic details of the interaction. A docked and simulated binding model was not proofed by site-directed mutagenesis, leaving open questions on details and redundance of the TIGIT binding site on Fap2. Finally, the structure comprised the ECD without the native *F. nucleatum* autotransporter domain, leaving open questions on the orientation of the Fap2 extracellular region relative to the membrane and how many of them might act together in the cellular context.

### RadD

The functional capabilities of *F. nucleatum* are further enhanced by another large type Va autotransporter adhesin, RadD. With ~350 kDa, RadD is almost as large as Fap2, and was shown to exhibit remarkable functional versatility. It is produced, amongst others, by Fnn strains ATCC 23726, ATCC 25586, and also by *F. nucleatum polymorphum* ATCC10953, but not by ATCC 12230.^39^ ^,^ ^96^ In Fnn ATCC 25586, the RadD protein had been initially misannotated as 2143 amino acids, and a new annotation of 3472 residues matches with experimental data that revealed a 370 kDa protein.^72^

RadD mediates bacterial coaggregation but also modulates carcinogenesis and immune evasion.^39^ ^,^ ^43^ ^,^ ^53^ The function of RadD is specifically linked to arginine- and lysine-inhibitable adherence,^39^ ^,^ ^97^ and it is a major contributor to polymicrobial interactions: Several studies showed that it facilitates binding of *F. nucleatum* to diverse microbes, including Gram-positive bacteria (*Streptococcus oralis, S. sanguinis, S. gordonii, S. mutans, S. cristatus, Actinomyces naeslundii, A. oris, Clostridioides difficile*),^39^ ^,^ ^40^ ^,^ ^96^ ^,^ ^98-100^ Gram-negative bacteria (*Aggregatibacter actinomycetemcomitans*),^97^ and fungi (*Candida albicans).* ^101^ For the gram-negative *Porphyromonas gingivalis*, RadD mediates co-aggregation with strain 4612,^28^ but not with strain PK1924.^97^

RadD from *F. nucleatum* ssp. *polymorphum* specifically binds to SpaP of *S. mutans*. The interaction site of SpaP for RadD appears to be distinct from regions otherwise known to be involved in binding to salivary glycoproteins, as binding of RadD to a C-terminally truncated version of SpaP that lacks the binding site for salivary agglutinin was not impaired.^100^ The authors hypothesized that the binding capability stems from highly variable regions, specifically between amino acids 285–625 or 1716–1811, that differ between fusobacterial strains.^100^ RadD of Fnn ATCC 23726 facilitates a cross-kingdom interaction with *C. albicans* that was shown to depend on the presence of FLO9, an adhesin-like mannoprotein, in the yeast cell wall.^101^ ^,^ ^102^ Interestingly, the coaggregation between *F. nucleatum* and *C. albicans* was sensitive to both mannose or arginine, suggesting the presence of binding sites for both molecules.^101^ The authors hypothesized that the binding of arginine to RadD could result in a conformational change of the mannose-binding pocket, thereby inhibiting coadherence. Sequence analysis of RadD identified a domain near its C-terminus with homology to a bacteriophage tail fiber, which may be involved in the interaction with the mannan residue of FLO9, similar to bacteriophage tail fibers binding to polymannose O-antigens.^101^ ^,^ ^103^

In addition to facilitating polymicrobial interactions, RadD was found to specifically target several eukaryotic host receptors. It has been identified as the bacterial ligand from Fnn ATCC 23726 for the inhibitory immune receptor Siglec-7, which is expressed on NK cells, and whose activation results in the inhibition of NK-mediated killing of cancer cells.^53^ The interaction with Siglec-7 was found to be subspecies-specific: whereas Fnn ATCC 23726, ATCC 25586, and the clinical isolate Fnn CTI-7 bound Siglec-7, two strains of *F. nucleatum subsp. polymorphum* showed a different binding behavior: strain ATCC 10953 bound Siglec-7, and strain 12230 did not due to the absence of *radD* in its genome.^53^ The interaction of RadD with Siglec-7 depends on R124 of Siglec-7, a residue known to be crucial for sialic acid-dependent ligand binding,^104^ and its mutation confirmed its critical role in the interaction.^53^ Consistent with the importance of an arginine-mediated contact, the RadD/Siglec-7 interaction was competitively inhibited by free L-arginine and L-lysine in a dose-dependent manner. Additionally, the monoclonal antibody S7.7 targeting Siglec-7 disrupted the interaction, whereas antibody K8 did not, indicating that RadD and S7.7 share overlapping binding sites.^53^ Interaction assays conducted with the same RadD suggest that it additionally binds to human IgA, which was also previously indicated for RadD from *F. nucleatum polymorphum* ATCC 10953.^53^ ^,^ ^96^ RadD from Fnn ATCC 23726 bound to both human IgA and Siglec-7 at distinct, non-overlapping sites, a dual-binding capability that has likely evolved independently, as RadD is only the second known bacterial protein, after the non-homologous GBS *β* antigen, to exhibit this function.^53^ ^,^ ^105^

A further human cell receptor for RadD was recently identified in a genome-wide transposon mutagenesis screen: CD147. RadD from both Fnn ATCC 23726 and *F. nucleatum subsp. polymorphum* ATCC 10953 was shown to bind HTC-116 colon cancer cells in a CD147-dependent manner.^43^ The binding of RadD from Fnn ATCC 23726 to CD147 activated the PI3K-AKT-NF-*κ*B-MMP9 signaling pathway, stimulating the release of matrix metalloproteases that promote CRC cell proliferation, migration, and invasion.^43^ ^,^ ^106^ Moreover, the abundance of the *radD* gene showed a correlation with both clinical grade of CRC and CD147 expression levels on tumor cells, implying that the RadD/CD147 interaction may also facilitate the enrichment of *F. nucleatum* within CRC tissues.^43^ Additionally, both arginine and lysine were shown to interfere with the RadD-dependent binding of *F. nucleatum* to CRC cells, similar to their effects in bacterial coaggregation.^39^ ^,^ ^43^ ^,^ ^97^ Here, it would be intriguing to investigate whether binding of RadD to CD147 is blocked directly by addition of these amino acids, or if they bind RadD and induce conformational changes that result in altered binding capabilities, similar to the previously mentioned mechanism that was postulated for the inhibition of interaction between *F. nucleatum* and *C. albicans.* ^101^ It also remains to be investigated which further *F. nucleatum* subspecies and strains, including clinical isolates, can bind CD147 via RadD.

Structure predictions and domain analyzes indicated that RadD from Fnn ATCC 23726 shares significant structural similarities with Fap2,^39^ ^,^ ^43^ ^,^ ^79^ though its tertiary structure remains unknown. Two different AlphaFold2 predictions of the ~370 kDa large RadD, both from Fnn ATCC 23726, resulted in the same secondary structure elements (i.e., *β*-helix) but with very different three-dimensional organization. Prediction of RadD in one piece as a full-length protein resulted in a circular organization of the protein in which the autotransporter is closely packed by the *N*-terminal part and a “head” domain.^43^ In contrast, prediction of the extracellular region in overlapping fragments resulted in a more linear, rod-shaped model, very similar to the Fap2 structure.^79^ The prediction quality metrics (i.e., pLDDT values) were high in both cases. This clearly illustrates limitations of prediction models of large and complex protein structures, which might result in misinterpretations of and discrepancies with biological data. In the case of RadD, the linear rod-shaped model appears more plausible due to the functional analogy to Fap2.

In addition, cryo-ET observations of the fusobacterial membrane suggested that RadD from Fnn ATCC 25586 has a similar overall length and rod-like shape to Fap2, as the two adhesins were abundantly present in the OM yet indistinguishable at the resolution of cryo-tomograms.^79^ Despite the comprehensive characterization of RadD binding to multiple receptors on various organisms and cell types, the exact molecular mechanism of these interactions remains unknown, and structural analysis of RadD in complex with its receptors is required to visualize molecular details of the interactions.

### CbpF

CbpF is a 150 kDa type Vc trimeric autotransporter adhesin (TAA) expressed by *F. nucleatum* and *F. vincentii.* ^55^ The protein comprises an *N*-terminal leader peptide, a surface-displayed passenger domain, and a 12-stranded C-terminal *β*-barrel domain in the outer membrane that is formed by three identical polypeptide chains together with four strands each.^107^ In contrast to the monomeric type Va ATs described above, the extracellular domain and the transmembrane *β*-barrel are made up together by the three identical CbpF monomers. TAAs are widespread pathogenic adhesion factors that promote host cell binding, invasion, and biofilm formation.^108^ Well-described examples from other bacteria include *Yersinia enterocolitica* YadA, *Moraxella catarrhalis* UspA1, *Burkholderia pseudomallei* BpaC, *Haemophilus influenzae* Hia, *Salmonella enterica* SadA, and *Bartonella henselae* BadA.^109-115^

**Figure 4.**
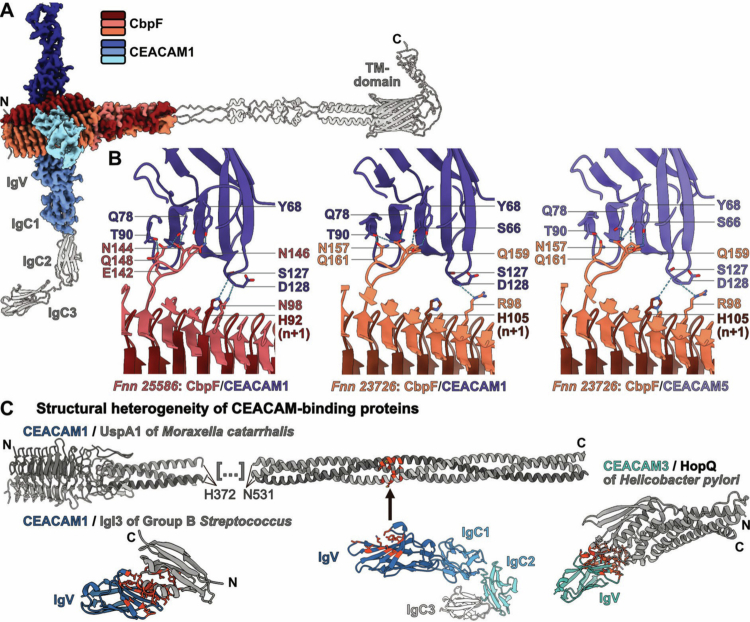
The adhesin CbpF. A: Structure of CbpF (Fnn ATCC 25586) in complex with CEACAM1, solved by cryo-EM to 2.7 Å resolution (PDB 9GH5, EMD-51347). The density map is colored in shades of red and blue corresponding to CbpF and CEACAM1 subunits, respectively. AlphaFold2 models of nonresolved regions are shown in transparent. B: Detail of Interaction sites of CbpF from Fnn ATCC 25586 (left) and Fnn ATCC23726 with CEACAM1 (PDB 9U94, middle) and CEACAM5 (PDB 9U93, right), respectively. H-bonds as identified by ChimeraX are indicated as blue dotted lines. C: Structure models of CEACAM-binding adhesins from the indicated bacteria (gray), for Igl3 and HopQ in complex with CEACAM1 and CEACAM3, respectively. Note that, whereas the binding sites of the CEACAMs are always situated at the GFC′C′ face (i.e., the dimerization interface) of the IgV domain, the binding site architectures of the adhesins are variable.

Recently, structural analysis of the CbpF trimer from Fnn ATCC 25586 by cryo-EM revealed that its ECD forms a homotrimeric *N*-terminal *β*-roll domain 64 Å in length and 44 Å in width, composed of 14 parallel *β*-strands per monomer. From this *β*-roll, a unique protruding loop emerges that is absent in the same regions of other bacterial TAAs. At the C-terminus, the *β*-roll is extended by an ~54 Å region of intertwined *α*-helices followed by an unresolved unstructured region conferring flexibility and potentially enabling multi-angle receptor engagement (Figure 4A).^116^ Shortly after, a structure of CbpF from Fnn ATCC 23726 revealed a very similar architecture, comprising a 6 Å longer *β*-roll region than in CbpF from Fnn ATCC 25586, while retaining the unique protruding loop (Figure 4B).^117^

CbpF utilizes CEACAM1 and CEACAM5 as receptors on human cells.^44^ ^,^ ^55^ ^,^ ^118^ CEACAMs are immunoglobulin (Ig-)like protein family members which form *trans*-homo- and -heterodimers that mediate adhesion across epithelial cells and prevent self-damaging autoimmune responses between epithelial and immune cells.^119-121^ Furthermore, they are overexpressed in colorectal cancer cells and strongly associated with colorectal cancer progression.^54^ ^,^ ^122^ Whilst *F. vincentii* ATCC 49256 was originally identified in a CEACAM1 binding screen, CbpF binding to CEACAM1 has also been identified in some, but not all, tested clinical isolates of *F. nucleatum*. Moreover, sequence analysis of different CbpF-like autotransporters in *F. nucleatum* subspecies *nucleatum, vincentii* and *polymorphum* showed length differences of the extracellular region, resulting in differential CEACAM1 binding.^55^

Structural and functional data show that CbpF from Fnn ATCC25586 binds CEACAM1 with high affinity via the *β*-roll protruding loop, which contains the key residues N144 and Q146, and H92 from the adjacent protomer chain (Figure 4A and B). Mutagenesis of either H92 or the loop residues abolishes binding, underscoring the significance of both interfaces.^116^ This also explains why only trimeric CbpF can bind CEACAM1.^55^ ^,^ ^116^ While other bacterial adhesins, e.g., *E. coli* fimbriae or CfaE, employ two different subunits to bind its receptor,^123^ ^,^ ^124^ CbpF uniquely requires two identical protomer chains for receptor binding. A further high-resolution structure of CbpF from Fnn ATCC 23726 shows that the key residues in the loop for binding and also the histidine in the neighboring subunit (H105, corresponding to H92 in CbpF from Fnn ATCC 25586) are retained.^117^

A further type Vc adhesin that binds CEACAM1 is UspA1 from *M. catarrhalis*. Despite structural similarities, UspA1 lacks the protruding loop of CbpF. Instead, CEACAM1 binds to the membrane-proximal coiled-coil region of UspA1 and not to the *β*-roll (Figure 4C). Yet, both adhesins target the GFC′C″ face of CEACAM1.^110^ Structurally unrelated to TAAs, the *Streptococcus agalactiae* *β*-protein IgI3 domain,^125^ AfaE from *Escherichia coli* ^126^ and HopQ from *Helicobacter pylori,* ^127^ also target the CEACAM1 GFC′C″ face. This pattern demonstrates that despite structural variability among bacterial CEACAM-binding proteins, the CEACAM1 target site remains highly conserved.

CEACAM1 interacts with CbpF through F63 and Q78, whereas CEACAM5 retains only the interactions involving Q78. However, CEACAM5 forms additional contacts with the adjacent CbpF subunit.^116^ ^,^ ^117^ The indispensability of Q78 for the interaction with CbpF was underscored by mutagenesis studies that abolished CEACAM1/5 binding, while mutagenesis of F63 abolished CEACAM1 binding.^55^ ^,^ ^117^ The architecture of the binding site provides the structural basis for the adhesive and immunoregulatory role of CbpF in CRC: CbpF specifically targets CEACAM1 and CEACAM5 while avoiding activating other CEACAM-family members, in particular CEACAM3. CEACAM3 activation promotes microbial clearance via its ITAM-like motif.^128^ The absence of Q78 in CEACAM3 reflects the evolutionary adaptation of CbpF to avoid this proinflammatory receptor, thereby enhancing immune evasion.

CbpF undergoes no conformational changes upon binding to CEACAM1, which is compatible with binding of up to three CEACAM1 molecules per CbpF homotrimer.^116^ Shen et al. expanded this observation proposing the “Velcro” model, where one CbpF trimer engages up to three CEACAM1/5 monomers or *cis*-dimers, whose binding partners recruit additional CbpF trimers to form tight multivalent networks.^117^ This underscores the hypothesis that the pathological influence of CbpF extends beyond immune evasion and additionally mediates *F. nucleatum* adhesion to cancer cells. Although the existing *in vitro* structures support the “Velcro” model, high-resolution *in situ* cryo-ET data will be essential to visualize and validate the CbpF/CEACAM1/5 assemblies under physiological conditions.

The physiological role of epithelial CEACAM1 is to suppress damaging autoimmune responses, and by overexpressing CEACAM1 and CEACAM5, tumor cells exploit this mechanism.^54^ ^,^ ^119^ ^,^ ^128^ *F. nucleatum* might even potentiate cancer-associated immunoresistance by adding further CEACAM1/5 binding sites through the trimeric CbpF. Functionally similar, UspA1 and Igl3 both confer adhesion and immune evasion for their respective pathogens.^110^ ^,^ ^125^ ^,^ ^129^ IgI3 and UspA1 both target CEACAM1 via Q78 while Igl3 additionally targets CEACAM5 as CbpF does. This makes Q78 of CEACAM1/5 a target that is shared by different adhesin morphologies and bacterial species for immune evasion and adhesion.

In summary, cryo-EM visualized key mechanistic details for the CbpF-CEACAM1/5 interaction, paving the way for structure-based binding inhibitor design that targets the unique loop of CbpF. The three existing structures have in common that they only visualize the *N*-terminal part of the ECD, despite nanodisc-embedded full-length CbpF has been applied for determination of the CbpF/CEACAM1 structure from *F. nucleatum* ATCC25585.^116^ Structure predictions suggest a flexible linker after the resolved region (Figure 4A), which might support a model in which the CbpF flexibility is utilized to catch receptors, analogous to a fishing rod. To describe and analyze such a mechanism, dynamic studies, e.g., using time-resolved single-molecule Förster resonance energy transfer spectroscopy, will be required. Another remaining open question is the functional role of CbpF within the diverse adhesin repertoire of *F. nucleatum*. In particular, how CbpF relates to the Gal-GalNAc- and TIGIT-binding adhesin Fap2 in CRC remains unclear, raising the possibility that *F. nucleatum* deploys distinct adhesins to target specific CRC subtypes or tumor microenvironments.

### Further *F. nucleatum* autotransporter proteins

In addition to the extensively studied adhesins described above, *F. nucleatum* has been reported to express further AT proteins with possible roles in CRC progression. A sequence analysis of the Fnn ATCC 23726 genome^81^ revealed 21 AT proteins, and nine of them were longer than 2000 residues.^79^ Therefore, likely further adhesins with high relevance in CRC analogous to RadD and Fap2 exist.

One of them is the apoptosis-inducing outer membrane protein 1 (AimI), a 197 kDa Fap2 homolog with 48% sequence identity in Fnn ATCC 23726. Aim1 has been shown to induce cell death in Jurkat cells.^52^ Although to our knowledge, no direct involvement of AimI in CRC has been reported so far, the immune cell toxicity suggests a role in immune evasion, as identified for Fap2 and CbpF. Whereas no experimental structure is available for Aim1, the high sequence identity to Fap2 suggests a comparable domain architecture, with an extended surface-exposed passenger domain that is made up of a *β*-helix. The molecular mechanism underlying AimI-mediated apoptosis, presumably encoded within the passenger domain, remains unresolved due to the absence of structural data and no identified receptors on host immune or epithelial cells.

**Figure 5.**
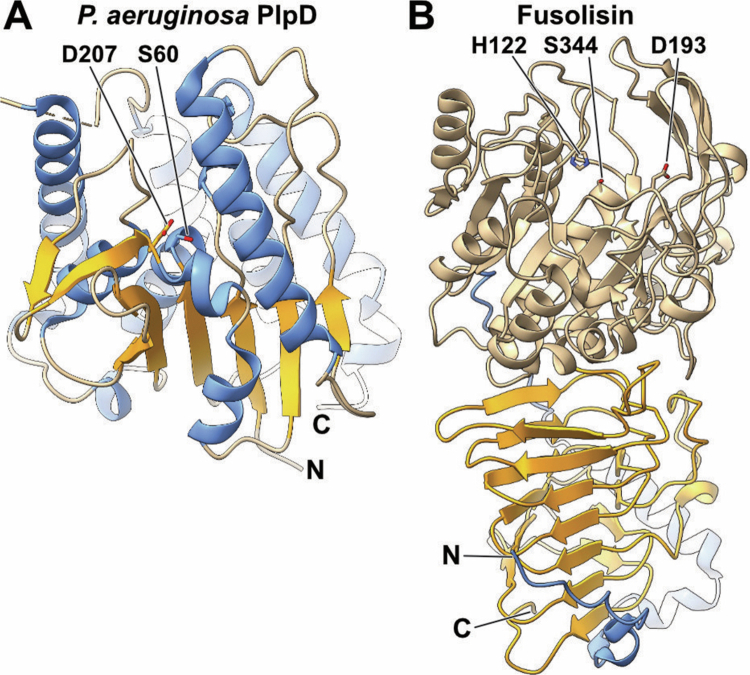
Structures of the phospholipase PlpD and the protease Fusolisin. A: Crystal structure of *P. aeruginosa* PlpD (residues 20—333; PDB 5FYA). The catalytic diad S60/D207 that is situated within a hydrophobic cavity in PlpD is indicated. B: Crystal structure of *F. nucleatum* ATCC25586 Fusolisin (residues 29—607; PDB 8AXG). The catalytic Ser-His-Asp triad in the protease domain is indicated.

The 85 kDa *Fusobacterium* phospholipase autotransporter (FplA) is to date the only known type Vd autotransporter protein in *F. nucleatum.* ^130^ The expression of the protein in Fnn strains ATCC 23726, ATCC 25586, 4_1_13, 4_8, and in *F. nucleatum polymorphum* (ATCC 10953) has been demonstrated under bacterial culture conditions via western blotting;^130^ however, the expression of FplA in vivo in biofilms and/or attached to host cells is yet unknown. The passenger domain of FplA remains membrane-associated and is not an adhesin like the other proteins described before, but instead exhibits phospholipase A1 activity. FplA belongs to the patatin-like phospholipase family, which adopt a conserved α/β-hydrolase fold with a catalytic Ser-Asp dyad.^131^ This phospholipase type has also been identified as a type V secreted protein in *Pseudomonas aeruginosa*, termed PlpD, whose crystal structure of the soluble part shows that the catalytic site is situated within a hydrophobic channel, covered by a flexible lid (Figure 5A).^132^ A homology model of FplA based on this structure suggests the same architecture and function as phospholipase because the catalytic site is conserved.^130^ The molecular basis of the proposed FplA-mediated intracellular invasion capabilities, e.g., escaping vacuoles and modulation of host autophagy and apoptosis,^130^ remains to be elucidated.

A further AT protein with an enzymatically active passenger domain is Fusolisin, a serine protease involved in immune downregulation. The 99 kDa type Va AT Fusolisin has been identified in subspecies *nucelatum*, *vincentii, and polymorphum*. The 55 kDa passenger domain carries the catalytic domain with the typical Asp-His-Ser catalytic triad.^133^ This passenger domain is processed in a strain-specific manner: Fusolisin from Fnn ATCC 25586 remains full-length (99 kDa), whereas Fusolisin from Fnn strains ATCC 23726 and FDC364, from subsp. *vincentii* (ATCC 49256), and from subsp. *polymorphum* (ATCC 10953 and ATCC 12230) was autoproteolytically cleaved to release the ~55 kDa *N*-terminal passenger domain.^133^ Immunomodulatory activity through Fusolisin is either mediated by the inactivation of host immune system mediators, such as IgA, the cleavage of extracellular matrix proteins such as fibronectin, fibrinogen, and collagens,^134^ or the inhibition of NK cells via the cleavage of activating receptors CD16, NKp44, and NKp46 which results in increased tumor cell survival.^135^ X-ray crystallography of Fusolisin from *F. nucleatum* ATCC25586 revealed the structure of a part of the passenger domain (Figure 5B), showing a subtilisin-like serine protease with a typical α/β-fold containing a 7-stranded parallel *β*-sheet (^136^ to be published). However, no structures of Fusolisin bound to its host substrates or immune effectors have been reported, leaving the molecular determinants of substrate specificity and immune modulation unresolved.

**Table 1.** Summary of structure and function of fusobacterial adhesins and AT proteins.

	Structure and type	Known host receptors	Functional outcomes
FadA	*α*-helical hairpin^1^; filament-formation via leucine chain motif	E-cadherin (CRC cells); VE-cadherin (endothelial cells)	Tumor colonization and CRC cell growth stimulation through *β*-catenin pathway; increase of endothelial permeability
Fap2	Type Va autotransporter, rod-shaped *β*-helical extracellular domain^1^	Gal-GalNAc (CRC cells); TIGIT (NK cells)	Tumor colonization; immune evasion of tumors colonized by *F. nucleatum*
RadD	Type Va autotransporter, rod-shaped *β*-helical extracellular domain^2^	CD147 (CRC cells) Siglec-7 (NK cells)	Tumor colonization and CRC cell growth stimulation through PI3K–AKT–NF–κB–MMP9 cascade; immune evasion of tumors colonized by *F. nucleatum*
CbpF	Homotrimeric type Vc autotransporter, *β*-roll extracellular domain^1^	CEACAM1; CEACAM5	Immune evasion of tumors; eventually tumor colonization
Aim1	Type Va autotransporter	unknown	Immune cell toxicity
FplA	Type Vd autotransporter^3^	Cleavage of phospholipids	Probable function in cell invasion
Fusolisin	Type Va autotransporter with subtilisin-like serine protease domain^1^	Degradation of cell matrix proteins (fibrinogen, fibronectin, collagen I and IV)	Probably NK cell inhibition and damage of periodontal tissue

^1^ Experimental structure.

^2^ Structure prediction.

^3^ Homology model.

## Conclusion and outlook

Here, we reviewed mechanistic details of *F. nucleatum* adhesins with known important roles in CRC progression, either through tumor cell adhesion (FadA), downregulation of the immune response (CbpF), or both (Fap2, RadD; Table 1). With cellular receptors and, to a certain extent, also the underlying intracellular pathways known already for years, the molecular mechanisms of binding have only recently begun to be elucidated. Cryo-EM and integrative modeling have provided unprecedented mechanistic insight into adhesin architecture and receptor recognition. They revealed a common structure‒function relationship of the autotransporter adhesins Fap2, RadD and CbpF: the extracellular parts are constructed as long “spacers” with receptor binding sites close to the most membrane-distal region. In addition, these spacers all show at least one structural motif that indicates flexibility. This architecture allows *F. nucleatum* to interact with the immune cell receptors at largest possible distance, and a site of flexibility allows for fishing rod-like movements. An important shared motif of all described fusobacterial adhesins despite their structural diversity is the multiplicity of binding. This allows weak individual adhesin–receptor interactions to be overcome through multivalency, with many individual binding events. The multiplicity of binding sites is achieved through oligomerization (CbpF), polymerization to filaments (FadA), or a high number of molecules on the surface (Fap2, RadD, as shown in ref.^79^). For the latter, the capacity of in vitro structural biology is limited to reliably visualize molecular details, especially when the interactions are highly transient and appearing in flexible regions of the proteins.

Despite the recent high-resolution structural analyzes of CbpF and Fap2, major mechanistic questions remain, amongst others regarding adhesin cooperation, regulation, and spatial organization on the host-*Fusobacterium* interface *in situ*. Future structural research should address these aspects through in situ structural biology (i.e., cryo-ET) at the cell surface and will be essential to understand how *F. nucleatum* adapts to tumor niches at different disease stages and to immune clearance.

Notably, surface exposure and structural specificity of adhesins make them attractive candidates for therapeutic targeting. Structure-guided inhibition of adhesin–host interactions will disrupt key protumorigenic pathways and overcome immune evasion without broadly perturbing the microbiome. However, key challenges have to be overcome in clinical translation, both with respect to *Fusobacterium nucleatum* itself and with respect to potential drug candidates. With many different adhesins, redundance of host‒pathogen interactions typically arises as soon as the target CRC cell type is positive for more than one receptor because CRC-associated *F. nucleatum* strains, such as recently discovered *F. nucleatum subspecies animalis* C1 and C2 produce several of the previously described adhesins.^80^ Therefore, combinations of binding inhibitors, eventually together with antibiotics, are probably required for successful therapeutic approaches. This has been successfully performed for the combination of FmlH and FimH in UPEC.^137^ Moreover, species-specificity of receptor binding limits the utilization of animal models, in particular for immunomodulation through Fap2. Another challenge for targeting fusobacterial adhesins in the tumor environment is target accessibility in the tumor microenvironment, which can be increased by utilization of delivery systems such as antibody-coated nanoparticles, which proved successful for a glycomimetic.^138^ Finally, it remains to be elucidated whether particular adhesion inhibitors are strain-specific or applicable throughout multiple CRC-associated strains.
